# Antimicrobial resistance in humans, livestock and the wider environment

**DOI:** 10.1098/rstb.2014.0083

**Published:** 2015-06-05

**Authors:** Mark Woolhouse, Melissa Ward, Bram van Bunnik, Jeremy Farrar

**Affiliations:** 1 Centre for Immunity, Infection and Evolution, University of Edinburgh, Ashworth Laboratories, Kings Buildings, Charlotte Auerbach Road, Edinburgh EH9 3FL, UK; 2 Wellcome Trust, Gibbs Building, 215 Euston Road, London NW1 2BE, UK

**Keywords:** biota, governance, Intergovernmental Panel on Climate Change, phylogenetics, reservoirs, sequencing

## Abstract

Antimicrobial resistance (AMR) in humans is inter-linked with AMR in other populations, especially farm animals, and in the wider environment. The relatively few bacterial species that cause disease in humans, and are the targets of antibiotic treatment, constitute a tiny subset of the overall diversity of bacteria that includes the gut microbiota and vast numbers in the soil. However, resistance can pass between these different populations; and homologous resistance genes have been found in pathogens, normal flora and soil bacteria. Farm animals are an important component of this complex system: they are exposed to enormous quantities of antibiotics (despite attempts at reduction) and act as another reservoir of resistance genes. Whole genome sequencing is revealing and beginning to quantify the two-way traffic of AMR bacteria between the farm and the clinic. Surveillance of bacterial disease, drug usage and resistance in livestock is still relatively poor, though improving, but achieving better antimicrobial stewardship on the farm is challenging: antibiotics are an integral part of industrial agriculture and there are very few alternatives. Human production and use of antibiotics either on the farm or in the clinic is but a recent addition to the natural and ancient process of antibiotic production and resistance evolution that occurs on a global scale in the soil. Viewed in this way, AMR is somewhat analogous to climate change, and that suggests that an intergovernmental panel, akin to the Intergovernmental Panel on Climate Change, could be an appropriate vehicle to actively address the problem.

## 1. Introduction

Human medicine is predominantly concerned with antimicrobial resistance (AMR) in human pathogens, but this is a very narrow view. Across all habitats, the total number of bacteria species alone may exceed one million [1] and only a tiny fraction of these, some 10–20 species such as *Mycobacterium tuberculosis* or *Staphylococcus pneumoniae*, are specialist human pathogens [2]. A somewhat larger number, several hundred species, are opportunistic pathogens that only cause human disease in certain circumstances, such as *Listeria* spp., *Campylobacter* spp. or *Staphylococcus aureus*. Hundreds more are part of the normal human microbiota, the majority of which are commensals that have not been linked to disease [3]. Many of the pathogenic bacteria (and probably many of the commensals too) occur not only in humans but also in other hosts, a wide variety of livestock and wildlife species (that is, they are zoonotic) or in the wider environment (sapronotic) or both, *Escherichia coli* being an obvious example [2].

**Figure 1.**
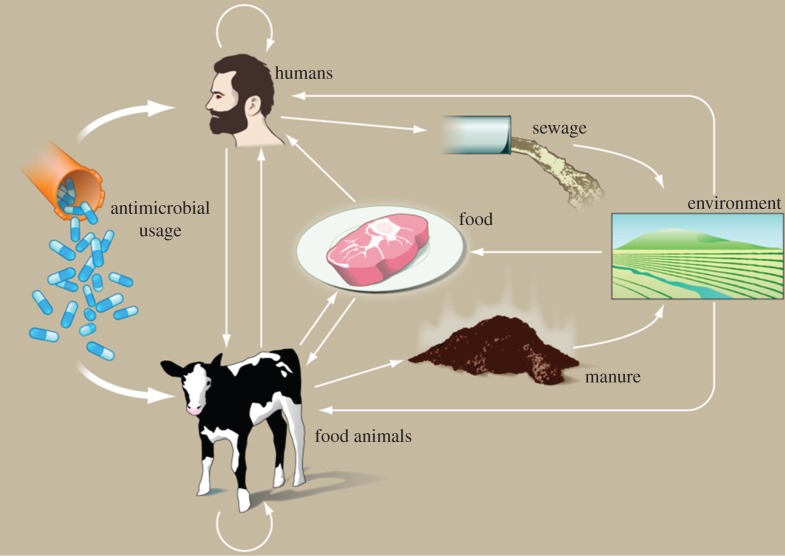
Diagrammatic representation of the routes of transmission of AMR between farm animals, the wider environment and humans. Reprinted with permission from [4] (Credit: P. Huey/Science).

The interplay between these different ecologies is especially important in the context of antibiotic resistance (figure 1). There are multiple links between the human, animal and environmental compartments that allow not only movement of the bacteria but also of mobile genetic elements (MGEs) and the drugs themselves [4]. The picture becomes even more complex when we also consider that antimicrobials affect more than one microbe species (the ‘multi-drug, multi-bug’ problem) and that resistance routinely moves between microbe species via MGEs.

Ideally, we need a sufficiently detailed, quantitative understanding of the dynamics of multiple bacteria, multiple drugs and multiple resistance determinants in multiple host and environmental compartments to make useful predictions. For example, we might reasonably ask: if we were to ban the use of an antimicrobial drug in farm animals what would be the impact on levels of resistance in human clinical cases? We still are a long way from being able to give robust answers to that kind of question. However, recent methodological advances point to ways to improve our understanding of the factors affecting levels of AMR in different host species. For example, phylogenetic analysis of bacterial genome sequences can be combined with epidemiological data by mapping traits such as AMR profile and host species onto bacterial phylogenies.

Here, we appraise four aspects of this challenging topic. First, we review the evidence that links AMR in different microbial populations in humans, other animals (especially livestock) and the wider environment. Second, we illustrate how state-of-the-art phylodynamic analysis can inform the evidence base. Third, we consider ways of reducing AMR within livestock populations. Finally, we reflect upon how we might achieve effective global governance of AMR that recognizes the importance of medicine, veterinary medicine and environmental microbiology.

## 2. Sources of resistance

### (a) Commensals

The advent of metagenomics has provided insights into the make-up of the human gut flora. One study found numerous resistance genes in the unculturable fraction, the so-called microbial ‘dark matter’ that comprises the bulk of the gut flora, but these were not homologous with resistance genes of clinical relevance [5]. By contrast, the culturable fraction contained numerous homologues of resistance genes in pathogenic bacteria. However, the direction of transfer between the commensal gut flora and the pathogens is uncertain.

### (b) Soil

The global biomass of microbes is enormous. A crude calculation consistent with published estimates [6] gives a value of approximately 50 tonnes of bacteria per person. Most of this biomass is found in soil, and soil is also the original source of the majority of antibiotics used in medicine and veterinary medicine [7]. Soil bacteria, and other soil microbes, have been producing antibiotics on a global scale for perhaps 2 billion years [8].

It is therefore not surprising that soil is also a major reservoir of AMR: resistance is likely to be as natural, widespread and ancient as antibiotic production. The relationship between resistance to naturally produced antibiotics in the soil and manufactured antibiotics in the clinic, however, remains unclear. For example, one recent metagenomics study found multiple examples of resistance genes in the soil that had 100% homology to those found in clinical isolates, across all major classes of antibiotics [9]. That study provides clear evidence for horizontal gene transfer between soil bacteria and pathogens, but it does not reveal in which direction(s) this has taken place. For example, the observation that resistance determinants for synthetic quinolones (*qnr* genes) can be detected in soil seems to indicate transfer from, not to, the clinic.

### (c) Farm animals

Industrial agriculture in its present form relies heavily on the widespread use of antimicrobials to improve animal health, welfare and productivity. Antimicrobials are used on livestock farms for a number of reasons: (i) as therapeutics; (ii) more commonly as metaphylactics, meaning that the presence of clinical illness in one animal triggers drug treatment of the whole herd or flock; (iii) prophylactics; and (iv) growth promotion. In Europe, antimicrobial usage is particularly high in intensively farmed species such as pigs and poultry and less so in extensively farmed cattle and sheep [10]. The list of antibiotics regarded as ‘critically important’ for farm livestock by the OIE (the World Organization for Animal Health) includes representatives of all major classes of antibiotics used in human medicine [11].

Growth promotion is a particularly controversial issue. It has been used extensively since the 1950s and has been reported to increase weight gain by up to 15–20%, a verysignificant effect [12]. The mechanism underlying growth promotion remains uncertain [13]; it works for antibacterials but not antifungals or antivirals, and it works for a variety of animal species including human children [14].

**Figure 2.**
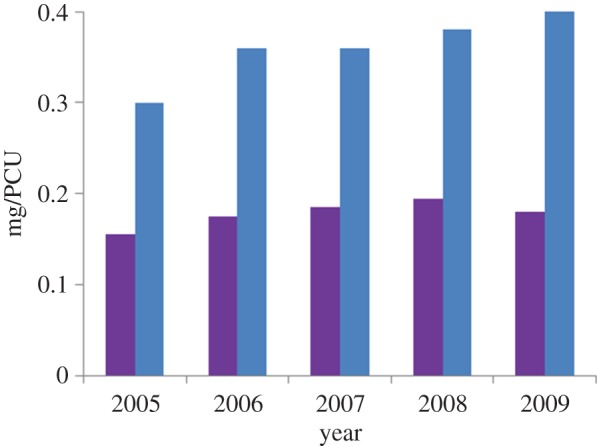
Sales of antibiotics for veterinary use in Europe, 2005–2009, for third and fourth generation cephalosporins (purple) and fluoroquinolones (blue). Units are milligram per population correction unit (=1 kg). In 2006, an EU-wide ban on the use of antibiotics as growth promoters was introduced. Data from [10].

A ban on the use of growth promoters was implemented throughout the EU in 2006. However, this has not led to any consistent decrease in antibiotic consumption (figure 2). Typically, the growth promoter ban has prompted compensatory increases in metaphylactic and prophylactic use. The result is that in Europe, the volume of agricultural usage of antibiotics continues to rival that of medical usage and in the USA (which recently introduced a voluntary ban on growth promoters), agricultural usage exceeds medical usage [15]. However, there have been some localized successes: for example, a more than 50% reduction in the usage of antibiotics (notably macrolides) in pigs was achieved from 1992 to 2008 in Denmark without any loss in productivity [16].

The key question regarding antimicrobial use in farm animals is whether and to what degree it poses a threat to human health. There are surprisingly few published studies which directly address this question. We know from numerous observational studies and surveillance reports that AMR is widespread in farm animals. Examples include: apramycin and ampicillin-resistant *E. coli* in newborn calves [17–20]; equally high levels of ampicillin resistance on organic farms [21]; and, more recently, the first reports of carbapenem-resistant enterobacteria in livestock [22]. However, such studies do not establish the direction of movement (if any) of resistance between human and livestock populations. Carbapenems, for instance, are not used in livestock so resistance was presumably imported from another, likely human, source. Bans on the use of avoparcin in animal feed in European countries in the 1990s were followed by reductions (by 75% in one German state) in levels of vancomycin-resistant enterococci in food products and in carriage in healthy humans [23]. But, in general, the benefits of reduced antimicrobial use in farm animals for human health remain unquantified.

## 3. Evidence from sequence data

Phylogenetic evidence has previously been used to determine the evolutionary origins of pathogen lineages in both human and livestock populations. A key feature of these kinds of analysis is that ancestral relationships may be inferred. For example, based on analysis of concatenated multi locus sequence type sequence data, it was concluded that MRSA lineage ST5 disseminated globally in poultry after a cross-species transmission from humans [24].

**Figure 3.**
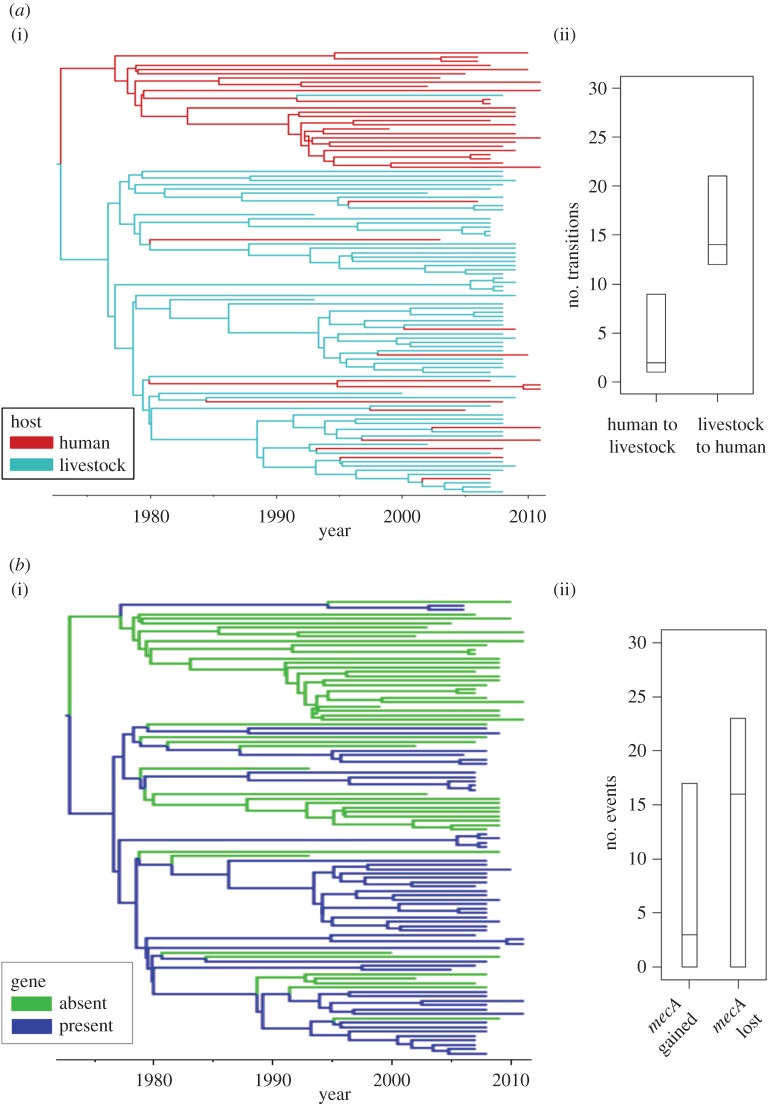
Discrete traits analysis of *S. aureus* CC398. Adapted and reprinted with permission from [26]. (*a*) Frequency of host jumps between human and livestock populations. (i) *Staphylococcus aureus* CC398 core genome BEAST maximum clade credibility tree with discrete-trait mapping by host. Branches are coloured according to inferred ancestral host (human or livestock). (ii) The inferred number of transitions between human and livestock hosts across 9000 BEAST phylogeny samples are plotted (95% highest posterior density intervals and medians shown as horizontal lines). (*b*) Frequency of gain and loss of *mecA*, a determinant of methicillin resistance for *S. aureus* CC398. (i) *Staphylococcus aureus* CC398 core genome BEAST maximum clade credibility tree with discrete-trait mapping for presence or absence of *mecA*. Branches are coloured according to inferred ancestral state (*mecA* absent or present). (ii) The inferred number of gains (transitions from absence to presence of *mecA*) or losses (transitions from presence to absence of *mecA*) across 9000 BEAST phylogeny samples are plotted (95% highest posterior density intervals and medians shown as horizontal lines).

The high genetic resolution provided by whole genome sequencing (WGS) provides a rich resource for inferring pathogen movements between host populations. Analysis of WGS data suggests that another MRSA lineage, CC97, has entered the human population from a livestock source on more than one occasion over the past 100 years [25]. A study of *S. aureus* CC398 from humans and livestock [26] has provided quantitative evidence that livestock-to-human jumps have occurred more frequently than human-to-livestock jumps over the evolutionary history of that lineage, consistent with CC398 being regarded as a livestock acquired MRSA (although with a separate clade associated with human-to-human transmission) (figure 3*a*). A similar study of *Salmonella* Typhimurium DT104 indicated multiple jumps between human and cattle populations over the past five decades as well as significant spread within human populations, which was unexpected as this is regarded as a food-borne pathogen [27].

In a novel analysis using WGS data, Ward *et al*. [26] were also able to quantify the gain and loss of specific antibiotic resistance determinants across the evolutionary history of MRSA CC398 (figure 3*b*). The analysis showed that methicillin resistance was gained and lost substantially more often than tetracycline resistance, and also indicated significant differences in numbers of gains and losses of two different tetracycline resistance determinants, *tetK* and *tetM*, consistent with their modes of inheritance.

Analysis of discrete traits upon phylogenies, e.g. as implemented in the BEAST software [28,29] and used in the aforementioned studies, provides a framework for inferring the nature and timing of character changes over the evolutionary histories of pathogens [30–32]. Such methods have great potential to improve our understanding of the factors influencing the gain and loss of resistance in natural populations, both through the movement of the pathogens themselves or the *de novo* appearance of resistance determinants. Their full power will be harnessed in the future through computational developments to allow larger numbers of bacterial whole genome sequences to be analysed, as well as by explicitly incorporating antimicrobial usage data and by analysing multiple traits (e.g. resistance phenotype and host species) simultaneously. Ultimately, however, this kind of analysis is entirely dependent on the availability of an appropriate selection of pathogen genomes. Sample collection needs to have spanned an appropriate time period and geographical range and, importantly, needs to cover all relevant host populations and reflect the phenotypic diversity of strains (e.g. in terms of resistance profile). In many instances, sampling is heavily biased towards humans, making the role of non-human reservoirs difficult or impossible to evaluate. This is one of several reasons to ensure that surveillance and monitoring are properly coordinated across different sectors and that sample collections are accompanied by appropriate metadata.

## 4. Managing resistance in farm animals

### (a) Surveillance

An important step towards assessing any threat to public health from AMR in farm animals is to determine levels of resistance in those populations. Yet there has been no systematic, international review of levels of AMR in farm animals. This aspect was ignored by the recent World Health Organisation (WHO) report on AMR globally [33] and the OIE and FAO (the international agencies with responsibility for livestock) have yet to conduct a similar exercise. Some countries, notably Denmark, have instigated coordinated reporting of AMR in humans and livestock [34] and the need for coordination is also recognized in the UK [35]. However, these remain exceptions.

National level reporting of AMR in farm animals typically relies on passive surveillance. For AMR in humans, alternatives to passive surveillance have been considered. These include open source reporting (e.g. ProMED or HealthMap [36]) and the identification of global hotspots where active surveillance might be targeted [37]. In principle, these kinds of approaches could be extended to farm animals, as has been suggested in the context of emerging zoonotic diseases in general [38].

### (b) Reducing antimicrobial usage in farm animals

Reducing the levels of antimicrobial consumption in farm animals has not proved straightforward, as the experience of the EU-wide ban on growth promoters illustrates (see §2*c*). Outside Europe, the adoption of voluntary codes and the development of guidelines for drug use, while welcome in themselves, seem unlikely to reduce consumption dramatically.

There may be some potential for more effective use of antimicrobials in farm animals, particularly if this generated tangible benefits in terms of reduced costs or improved productivity. These include the same approaches that have been proposed for human medicine, such as overkill strategies, combination therapies and drug reuse and recycling (e.g. [39]). Again as for humans, there would be obvious advantages of rapid diagnosis of bacterial infections and real-time profiling of resistance determinants using whole genome sequence data (e.g. [40,41]) to determine treatment strategies more quickly and accurately.

A complete ban on the use of antimicrobials in farm animals would inevitably have serious repercussions for animal health, welfare and productivity, and consequently on food prices. At present, it would be extremely hard to justify such an action in terms of the expected benefit to human health, given that the evidence for a direct link is so inconclusive (see §2c). However, reduced antimicrobial consumption in farm animals could form part of a coordinated strategy across the different sectors [35]. Any adverse effects of this on the agricultural industry would be at least partially alleviated if viable alternatives to antimicrobials were available.

### (c) Alternatives to antimicrobials for farm animals

The range of potential alternatives to antimicrobials in farm animals [42] is, for the most part, the same as for human medicine. There are currently a number of prebiotics and probiotics available, though their efficacy is unclear and likely variable. Mixing the two has also been proposed, so-called ‘synbiotics’. Phage therapy can be effective, for example against *Salmonella* Typhimurium in poultry and pigs, although this requires rapid selection and administration of the phage and high bacterial loads [42]. It may be possible to use purified phage lysins directly rather than the phage itself, thus precluding unintended transfer of genetic material from the phage. However, none of these possibilities is close to being available for commercial use on a global scale against the full spectrum of microbial disease in farms animals.

A more immediately practical proposition may be to expand the range of vaccines available for veterinary use. Although vaccines are already available against many of the major viral diseases of livestock, there is currently limited routine use of vaccines that protect against bacterial infection and disease. Even when it is available, a vaccine is not automatically adopted by producers: for example, one trial of a live oral *Lawsonia* vaccine in pigs resulted in both 80% lower consumption of oxytetracycline and increased productivity [43], but the vaccine is not widely used. As long as antibiotics are still available and effective, there is arguably little commercial incentive either to use existing or to develop new antibacterial vaccines for farm animals.

A longer term vision for reducing antimicrobial usage in farm animals might include the use of livestock that are genetically resistant to infection or disease, likely through the use of genetic modification technologies. One example of early progress in this direction comes from the development of transgenic chickens that do not transmit avian influenza [44].

Overall, however, it is clear that there would need to be considerable investment in research and development before any of the above approaches to disease control in farm animals become effective replacements for antimicrobials.

## 5. Discussion and recommendations

As recently pointed out [45], the challenge of tackling AMR has a number of parallels with the challenge of tackling another twenty-first century crisis, climate change. Both AMR and climate change are natural processes operating on a global scale that human activity has influenced only in the past half-century or so. However, there are also some important differences. In the context of climate change, alternatives to the burning of fossil fuels are already available and are beginning to be adopted on a significant scale. By contrast, as pointed out in §4c, alternatives to antimicrobials are not so far advanced. In the context of climate change, evidence-based targets for reductions in carbon dioxide emissions have been developed and agreed. By contrast, there are no agreed targets for reductions in antimicrobial usage, nor does the evidence base exist that could be used to set them. Humans currently produce, use and misuse an estimated 175 000 tonnes of antibiotics per year [15], but it is not even clear whether *any* level of antimicrobial usage is sustainable in the long term; many regard the emergence of AMR as inevitable. Finally, in the context of climate change, an international body, the Intergovernmental Panel on Climate Change, has been set up to marshal the scientific evidence and inform policy-making. There is currently no equivalent for AMR.

The case for an intergovernmental panel, or similar initiative, to tackle AMR comes from the realization that the problem is global and cross-sectorial, encompassing medicine, agriculture and the wider environment and so cutting across the remit of multiple international agencies including the WHO, OIE/FAO and UN [45]. There is a clear need for research input from a range of disciplines, not only clinical and veterinary medicine, epidemiology, microbiology and pharmacology, but also health economics, international law and social science. However, effective action on AMR will require a coordinated response from governments, industry and international agencies as well as scientists. That action will need to involve and will affect clinicians, pharmacists, patients, veterinarians and farmers, all of whom have contributed to the current AMR problem and all of whom will be part of a long-term solution. The most immediate need is to develop strategies for improved antimicrobial stewardship (in both human medicine and industrial agriculture), reinvigorate the antimicrobial drug pipeline and to develop effective and sustainable alternative approaches to tackling microbial disease in both humans and livestock.
